# A patterns of care analysis of hyperthermia in combination with radio(chemo)therapy or chemotherapy in European clinical centers

**DOI:** 10.1007/s00066-022-01980-9

**Published:** 2022-08-29

**Authors:** Adela Ademaj, Paraskevi D. Veltsista, Dietmar Marder, Roger A. Hälg, Emsad Puric, Thomas B. Brunner, Hans Crezee, Dorota Gabrys, Martine Franckena, Cihan Gani, Michael R. Horsman, Robert Krempien, Lars H. Lindner, Sergio Maluta, Markus Notter, Griseldis Petzold, Sultan Abdel-Rahman, Antonella Richetti, Andreas R. Thomsen, Pelagia Tsoutsou, Rainer Fietkau, Oliver J. Ott, Pirus Ghadjar, Oliver Riesterer

**Affiliations:** 1 Centre for Radiation Oncology KSA-KSB, Cantonal Hospital Aarau, Aarau, Switzerland; 2 Doctoral Clinical Science Program, Medical Faculty, University of Zurich, Zurich, Switzerland; 3 Department Radiation Oncology, Charité Universitätsmedizin Berlin, Berlin, Germany; 4 Institute of Physics, Science Faculty, University of Zurich, Zurich, Switzerland; 5 Department of Radiation Oncology, University Hospital Magdeburg, Magdeburg, Germany; 6 Department of Radiation Oncology, Cancer Center Amsterdam, Amsterdam UMC, University of Amsterdam, Amsterdam, The Netherlands; 7 Department of Radiation Oncology, Maria Sklodowska-Curie National Research Institute of Oncology, Gliwice branch, Poland; 8 Department of Radiation Oncology, , Erasmus MC Cancer Institute, Rotterdam, The Netherlands; 9 Centre for Radiation Oncology, University Hospital Tübingen, Tübingen, Germany; 10 Department of Clinical Medicine and Experimental Clinical Oncology, Aarhus University Hospital, Aarhus, Denmark; 11 Department of Radiation Oncology, Helios Hospital Berlin-Buch, Berlin, Germany; 12 Department of Medicine III, University Hospital, LMU Munich, Munich, Germany; 13 Department of Hyperthermia, Serena Medical Center, Padova, Italy; 14 Department of Radiation Oncology, Lindenhof Hospital, Bern, Switzerland; 15 Department of Radiation Oncology, Hospital Chemnitz, Chemnitz, Germany; 16 Radiation Oncology Clinic, Oncology Institute of Southern Switzerland, Bellinzona, Switzerland; 17 Department of Radiation Oncology, University Hospital Freiburg, Freiburg, Germany; 18 German Cancer Consortium (DKTK), Partner Site Freiburg and German Cancer Research Center (DKFZ), Heidelberg, Germany; 19 Department of Radiation Oncology, Geneva University Hospitals, Geneva, Switzerland; 20 Department of Radiation Oncology, University Hospital Erlangen, Friedrich-Alexander-University Erlangen-Nürnberg (FAU), Erlangen, Germany; 21 University of Zurich, Zurich, Switzerland

**Keywords:** Thermometric parameter, Thermal dose, Time interval, Treatment sequence, Treatment standardization

## Abstract

**Purpose:**

The combination of hyperthermia (HT) with radio(chemo)therapy or chemotherapy (CT) is an established treatment strategy for specific indications. Its application in routine clinical practice in Europe depends on regulatory and local conditions. We conducted a survey among European clinical centers to determine current practice of HT.

**Methods:**

A questionnaire with 22 questions was sent to 24 European HT centers. The questions were divided into two main categories. The first category assessed how many patients are treated with HT in combination with radio(chemo)therapy or CT for specific indications per year. The second category addressed which hyperthermia parameters are recorded. Analysis was performed using descriptive methods.

**Results:**

The response rate was 71% (17/24) and 16 centers were included in this evaluation. Annually, these 16 centers treat approximately 637 patients using HT in combination with radio(chemo)therapy or CT. On average, 34% (range: 3–100%) of patients are treated in clinical study protocols. Temperature readings and the time interval between HT and radio(chemo)therapy or CT are recorded in 13 (81%) and 9 (56%) centers, respectively. The thermal dose quality parameter “cumulative equivalent minutes at 43 °C” (CEM43°C) is only evaluated in five (31%) centers for each HT session. With regard to treatment sequence, 8 (50%) centers administer HT before radio(chemo)therapy and the other 8 in the reverse order.

**Conclusion:**

There is a significant heterogeneity among European HT centers as to the indications treated and the recording of thermometric parameters. More evidence from clinical studies is necessary to achieve standardization of HT practice.

**Supplementary Information:**

The online version of this article (10.1007/s00066-022-01980-9) contains supplementary material, including the survey’s questions, which were designed using different functionalities within the SurveyMonkey platform.

## Introduction

Hyperthermia (HT) in combination with radio(chemo)therapy and/or chemotherapy (CT) is an established treatment strategy for cancer. However, despite a multitude of preclinical and clinical studies that clearly demonstrate the potential of HT to enhance the efficacy of radio(chemo)therapy or CT, its availability in clinical centers remains limited. Reasons for this are the lack of reimbursement in many countries, the labor-intensive nature of HT delivery, the lack of up-to-date clinical trials where HT is combined with modern radiotherapy (RT) and drugs, and the lack of standardized treatment protocols and high-quality guidelines. HT deserves further clinical development based on decades of evidence that show improved clinical outcomes when HT is used in combination with radio(chemo)therapy or CT, such as for recurrent breast cancer, bladder cancer, cervical cancer, head and neck cancer, soft tissue sarcoma, and melanoma [1]. In addition, rectal [2], anal [3], pancreatic [4], and pediatric cancers [5] are indications where patients might benefit from including HT in the treatment strategy and clinical research is ongoing. HT is also in development for new indications, such as in combination with salvage radiotherapy in patients with recurrent prostate cancer after prostatectomy [6].

There are a variety of HT devices and techniques available, including local superficial, locoregional/regional deep, and moderate whole-body HT using electromagnetic, ultrasound, hyperthermic perfusion, and conductive heating [7]. The availability of techniques in clinical centers might influence which indications are actually treated.

Preclinical findings clearly demonstrate that the effectiveness of HT depends on thermometric parameters [8–11] such as temperature metrics, heating duration, number of sessions, time interval, and sequencing of HT and radio(chemo)therapy or CT. Unfortunately, the clinical evidence still lags behind the preclinical evidence. Consequently, clinical treatment protocols differ in many centers. The European Society for Hyperthermic Oncology (ESHO) published, and regularly updates, quality assurance guidelines for superficial and regional deep HT. So far, only a few reference temperature metrics are included in the ESHO guidelines and only for superficial cancer sites [12]. Furthermore, the German Atzelsberg Research Group recommended keeping records of temperature metrics and heating duration in their guideline about clinical application and documentation of regional deep HT, but did not specify reference values [13]. This illustrates the lack of robust reference values for many temperature metrics.

The European Hyperboost consortium (https://www.hyperboost.eu/) focuses on advancement of personalized HT treatment. Within Hyperboost, preclinical, physical, and clinical scientists join forces to boost HT research. One of the objectives of Hyperboost is the definition of optimal thermometric parameters for HT treatment by retrospectively analyzing large patient cohorts and correlating recorded thermometric parameters with clinical outcome. Prospective randomized studies will be designed for the validation of parameters derived from these analyses.

Before undertaking the retrospective analyses, the Hyperboost clinical group decided to perform a pattern of care analysis among the major European HT centers. The aim of this survey was to learn about indications treated, HT techniques used, and thermometric parameters stored in the European HT centers. To our knowledge, this is the first pattern of care analysis about the practice of HT in combination with other cancer treatments at the European level.

## Materials and methods

### Survey design

The survey was performed online using a commercially available platform (SurveyMonkey; www.surveymonkey.co.uk). The questions were developed with experienced researchers in the field of HT from the clinical Hyperboost group including authors from Aarau, Berlin, and Erlangen. The survey was started in June 2021. It comprised 22 single questions, 12 multiple-choice questions, 3 multiple-choice with one comment box, and 7 free-text questions. The survey questions are shown in the supplementary file.

This was a descriptive survey addressing two main topics: 1) clinical indications treated with HT in combination with radio(chemo)therapy or CT and 2) thermometric parameters recorded during patient treatment. In addition, the last question of the survey required confirmation from the respondents as to their future participation in a pooled retrospective analysis of thermometric parameters within the Hyperboost project.

### Survey distribution

Altogether, 24 clinical centers located in Europe were approached that apply superficial, deep, and whole-body HT for treating cancer patients. From each center, the head of the HT facility was contacted and invited to participate. Participants were only allowed to answer the survey once.

The HT centers included in the survey are the clinical centers of the Hyperboost consortium (Cantonal Hospital Aarau, Charité Universitätsmedizin Berlin, University Hospital Erlangen, Erasmus University Medical Center, Amsterdam University Medical Centers, University Hospital Aarhus) and, in addition, other major European HT centers (Ludwig-Maximilians-University Hospital, University Hospital Freiburg, University Hospital Tübingen, University Hospital Düsseldorf, Geneva University Hospitals, Lindenhof Hospital, Helios Hospital Berlin-Buch, Helios Hospital Bad Saarow, Hospital Chemnitz, Center for Radiation Therapy and Radiation Oncology Bremen, University Hospital Magdeburg, Medical Center Bad Trissl GmbH & Co., Ordens Medical Center Linz, Serena Medical Center, Hospital Santa Maria delle Croci, Greater Poland Cancer Centrum, Maria Sklodowska-Curie National Research Institute of Oncology and University Hospital Attikon).

### Statistical analysis

This is a descriptive report and percentages were used to describe the categorical variables. The mean ± standard deviation (SD) was used for continuous variables. The descriptive-based analysis and graphical plots were performed using IBM SPSS software (version 24.0, IBM, Armonk, NY, USA) and R programming (version 4.03).

## Results

### Response to survey and number of patients treated per center

In total, 17 clinical centers located in Switzerland, Germany, the Netherlands, Denmark, Italy, and Poland responded to the survey, yielding a good response rate of 71%. Sixteen out of 17 clinical centers also confirmed their participation in a pooled retrospective data analysis of thermometric parameters. The answers from Aarhus University Hospital were excluded because they stopped treating patients with HT many years ago.

**Fig. 1:**
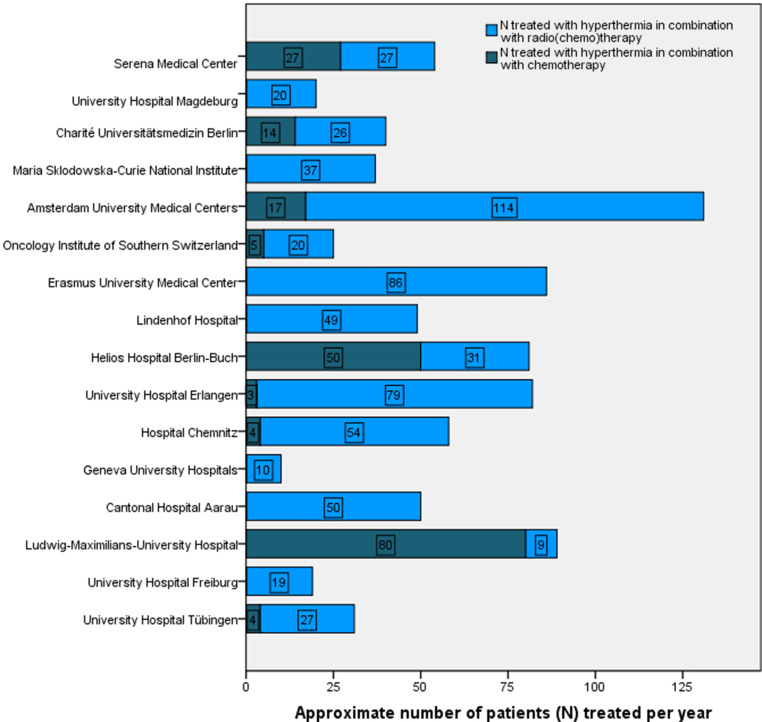
Approximate number of patients (N) treated yearly per clinical center with hyperthermia (HT) in combination with chemotherapy (in total 205) and with HT in combination with radio(chemo)therapy (in total 637)

In total, 637 patients were treated per year in the 16 clinical centers. The number of patients treated with HT differs among clinical centers. Overall, European HT centers treat an average of 40 ± 30 patients per year with HT in combination with radio(chemo)therapy, and 13 ± 22 patients are treated with HT in combination with CT. Fig. 1 illustrates the approximate frequency of patients treated per year in the 16 European clinical centers.

### Clinical indications and number of patients treated per indication

The most common tumors treated with HT in combination with radio(chemo)therapy were, in decreasing frequency, recurrent breast cancer 37% (235/637), cervical cancer 16% (104/637), sarcoma 13% (82/637), rectal and bladder cancer 6% (37/637), anal cancer 5% (31/637), head and neck cancer 3% (11/637), pancreatic cancer 3% (11/637), and one pediatric cancer patient. The remaining patients (13%, 83/637) were reported as “other patient cohorts” treated with HT in combination with radio(chemo)therapy. An average of 34% (range: 3–100%) of the total number of patients were treated in clinical study protocols.

**Fig. 2:**
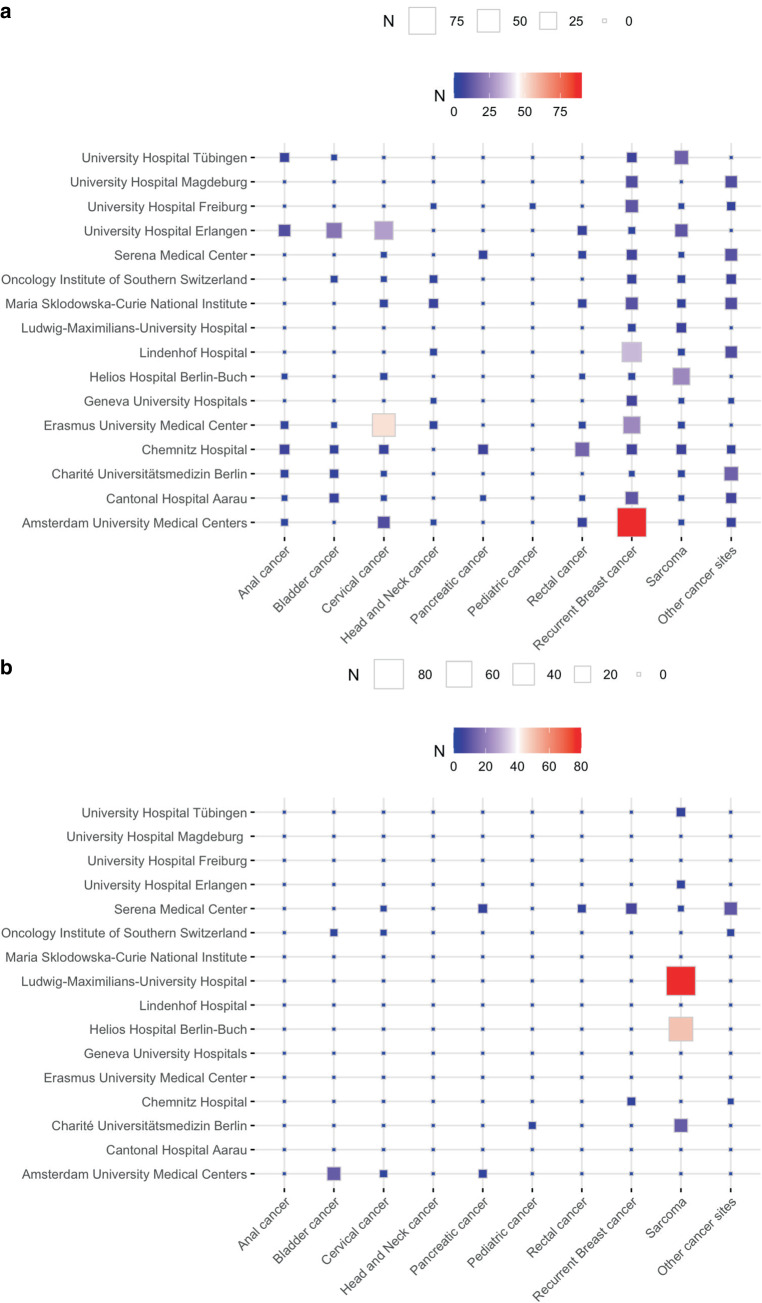
Approximate number of patients (N) treated for different indications per year with hyperthermia in combination with **a** radio(chemo)therapy and **b** chemotherapy

The major clinical indication treated with HT in combination with CT alone was sarcoma 73% (150/205), followed by bladder cancer 7% (14/205), recurrent breast cancer 5% (10/205), pancreatic cancer 3% (7/205), cervical cancer 2% (4/205), rectal cancer 2% (3/205), and 2 pediatric cancer patients. The other unspecified clinical indications treated with HT in combination with CT included 7% (14/205) of patients. Fig. 2a, b show the approximate number of patients treated yearly for different cancer types per clinical center.

### Hyperthermia techniques and type of treatment planning

We hypothesized that the availability of different HT devices might contribute to the variation in indications between clinical centers. However, there was actually little correlation between the type of HT device and the clinical indications treated. All 17 centers applied superficial HT and 10 centers deep HT, of which only one center (Serena Medical Center) used only the capacitive technique. Table 1 summarizes the frequency of HT techniques used in clinical centers for treating superficial and deep-seated tumors.

**Table 1:** The frequency of superficial, deep, and moderate whole-body HT techniques in 16 European clinical centers

HT techniques	Clinical centers (total *n* = 16)
*Superficial HT*	
Superficial radiative	13
Superficial infrared	4
*Deep HT*	
Deep radiative	9
Deep capacitive	2
*Moderate whole body*	1

*HT *hyperthermia

With regard to treatment planning techniques, 11, 1, and 4 clinical centers reported that they create geometric-based, simulation-based, and both geometric- and simulation-based plans for patient treatment, respectively. It can be concluded that the patient treatment plans in 15/16 clinical centers are comparable in quality to each other.

### Thermometric parameters

The second objective of the survey was to assess the availability and variability of thermometric parameters between clinical centers. The list of potentially relevant hyperthermia parameters includes temperature metrics, heating duration, thermal dose, number of HT sessions, and time interval between and sequencing of HT and the other cancer treatment, e.g., radio(chemo)therapy or CT. To keep the survey manageable for the respondents, questions only addressed the following thermometric parameters: temperature metrics, thermal dose, time interval, and sequencing.

#### Temperature metrics and thermal dose

All experts (100%) confirmed that they measure the temperature of normal tissues and tumor tissues (when clinically reasonably possible) during HT treatment. A record of temperature metrics, i.e., minimum temperature achieved in the target volume (T_min_) or temperature achieved in 50% of the target volume (T_50_), is kept in 81% (13/16) of centers. However, the well-known concept of thermal dose CEM43°C, proposed by Sapareto et al. [14], is used only in 31% (5/16) and the new TRISE thermal dose concept proposed by Franckena et al. [15] is computed in none of the clinical centers.

#### Time interval and sequencing

The time interval in hours and the sequencing of HT in combination with radio(chemo)therapy or CT vary between clinical centers.

The time interval between HT and radio(chemo)therapy or HT and CT is recorded in 9/16 (56%) and in two out of three centers, respectively. The sequencing between HT and radio(chemo)therapy is complex, as there is little or contradictory clinical evidence about its effect on treatment outcome. This survey showed that 50% (8/16) of clinical centers treat patients with HT prior to radio(chemo)therapy and 50% apply the reverse sequence.

The order of treatment modalities also matters when HT is combined with CT. In fact, the complexity of sequencing HT and CT is likely to be even higher in comparison to radio(chemo)therapy, because it is influenced by type, biological effect, and concentration of chemotherapeutic drugs. In 6 out of 9 centers that combine HT with CT only, both modalities are given simultaneously, and in three centers, sequentially. Of these three centers, in two CT is given after HT and in one clinical center the order is reversed. In addition, in three centers that treat patients with combined radiochemotherapy and HT, the RT part may be given sequentially and the CT part simultaneously with HT.

## Discussion

The results of this work show significant heterogeneity in the clinical indications treated yearly in 16 major European HT clinical centers as well as in the status of recorded thermometric parameters. The most common cancer entities treated with HT in combination with radio(chemo)therapy are recurrent breast cancer, cervical cancer, sarcoma, rectal, and bladder cancer. For the combination of HT and CT, the most frequent indications are sarcoma, bladder cancer, and recurrent breast cancer. The Amsterdam University Medical Center and the University Medical Center of Munich treat the highest annual number of patients with HT in combination with radio(chemo)therapy or CT, respectively.

Although the outcomes of this survey highlight the need for an international consensus with regard to recording of thermometric parameters, there were actually areas of agreement for guidance of the HT treatment process. All 16 participating centers confirmed that they monitor temperature during HT sessions when it is possible. In clinical practice, the temperature is monitored and measured by invasively placing or inserting probes within or near the target volume [12, 13]. However, this procedure is not feasible for all cancer sites, e.g., in the case of bone metastases, as the probes cannot be inserted invasively within or close to the target volume in all cases [16]. Non-invasive techniques to measure temperature, e.g., by use of magnetic resonance imaging, are currently in development, and so far only available in few HT centers [17]. The results of our survey indicate that 13 out of 16 (81%) clinical centers maintain a register of temperature data, particularly temperature metrics, as strongly recommended in the ESHO guidelines [12, 13]. The recording of temperature data is important because it enables assessment of the quality of heat delivery by computing temperature metrics such as minimum, average, or maximum temperature achieved. In addition, the temperatures achieved in 10%, 50%, and 90% of the tumor volume should also be recorded [12, 13], because these metrics have been shown to significantly correlate with treatment response [18]. In a planned multicentric analysis, the temperature metrics data from the 16 clinical centers will be collected and analyzed to find their relationship to the treatment response for different cancer sites.

Measuring the time between HT and the other treatment modality is an important parameter, because the time interval influences the radiosensitization and chemosensitization effects induced by HT [9]. Preclinical studies suggest that the strongest sensitization effects of HT are achieved when HT is delivered simultaneously with RT and CT and only additive effects were observed for a longer than 4‑h time interval between both modalities [8, 19, 20]. This is supported by a recent study in patients with recurrent breast cancer where an extremely short time interval between HT and hyopfractionated RT resulted in excellent complete and partial response rates [21]. Concerning the application of very short time intervals when deep HT is combined with radio(chemo)therapy, such an approach is almost impossible due to technical and logistical obstacles. Two retrospective clinical studies actually analyzed the effects of short and long intervals (shorter and longer than 4 h) between HT and radio(chemo)therapy for treatment of patients with cervical cancer and reported contradictory outcomes [22, 23]. In our survey, 9 of 16 clinical centers record the time interval between HT and radio(chemo)therapy or sequential CT. In addition to the conflicting data, logistical challenges in daily routine, such as the delivery of HT and other modalities in different institutions, influence the duration of the time interval achieved in individual patients and clinical centers.

Interestingly, only five (31%) clinical centers compute the thermal dose as CEM43°C during patient treatment. Among thermometric parameters, CEM43°C is considered to be one of the most promising because it has been shown to be prognostic in patients with superficial and deep-seated tumors [24, 25]. In contrast, other authors have expressed skepticism about the importance of using CEM43°C as a measure of thermal dose [26]. The issues raised in the latter study could be one of the reasons why only five clinical centers routinely use this thermal dose concept for assessment of HT treatment.

A well-known clinical parameter is the sequencing of HT with other cancer treatments. With regard to the combination of HT and radio(chemo)therapy, there exists little clinical evidence about the optimal sequencing of the two modalities. In clinical practice, the sequencing is often performed in analogy to preclinical studies [8, 19]. To date, no prospective clinical study has been designed to evaluate the effects of sequencing HT with other cancer treatments for any cancer site. The lack of clear evidence about the sequencing of HT is reflected by the results of this survey, in which 50% of clinical centers treat patients with HT prior to radio(chemo)therapy and 50% treat patients in the reverse order, independent of the cancer site.

Little variability was observed in our survey with regard to HT techniques and types of treatment planning. The development of a modern treatment planning system that enables both high-quality prescription of HT and recording of relevant thermometric parameters is a major research objective of the Hyperboost project. Standardized recording of hyperthermia data is a prerequisite for the analysis of large patient cohorts. So far, current oncology information systems cannot store HT treatment data in an automated manner. This has led to the current situation whereby HT data are heterogeneously stored in the European centers: raw data from the device database (6/16), spreadsheet software (4/16), Elekta (Crawley, UK) care management software (2/16), or paper based (3/16).

Standardization of HT treatment is challenging because of the relatively low numbers of patients treated with HT for most indications in Europe, as well as the low patient numbers included in prospective clinical studies. Therefore, clinical research should focus on multicenter retrospective analyses as well as well-designed prospective patient registries, the latter including a comprehensive and strict recording of HT parameters. With a proper study design, including a relevant and well-defined clinical endpoint and using multivariable modeling, such retrospective and prospective multicenter data analyses could probably provide enough clinical evidence for treatment standardization. More challenging and thus probably not the first option is conducting randomized clinical trials for optimization of thermometric parameters, again due to the difficulty of recruiting enough patients and because randomized studies rather focus on clinical endpoints and novel treatment combinations than on technical aspects of HT treatment delivery. A more innovative approach would be to conduct translational research phase I or II studies, where a biological read out could indicate the advantage of a specific set of treatment parameters, e.g., an optimal scheduling of RT and HT.

## Conclusion

The clinical indications treated with HT in combination with radio(chemo)therapy or CT differ considerably among 16 European clinical centers. The use of a variety of HT treatment protocols in these clinical centers also includes inconsistent measurement and recording of thermometric parameters. All centers reported generating geometric-based treatment plans. Future prospective data collection should include standardized recording and analysis of thermometric parameters. This survey forms the basis for retrospective and prospective European studies that will contribute to the standardization of HT treatment delivery.

## Supplementary Information

Hyperthermia boosting the effect of radiotherapy HYPERBOOST project: Survey questionnaire
