# Supplementary material for: A patterns of care analysis of hyperthermia in combination with radio(chemo)therapy or chemotherapy in European clinical centers
Source: Strahlenther Onkol. 2022 Aug 29;199(5):436–44. doi: 10.1007/s00066-022-01980-9 (PMC10133066; doi:10.1007/s00066-022-01980-9)
Supplement: Supplementary file 1 — Hyperthermia boosting the effect of radiotherapy HYPERBOOST project: Survey questionnaire [file 66_2022_1980_MOESM1_ESM.pdf]

Hyperthermia boosting the effect of Radiotherapy
HYPERBOOST project: Survey

* 1. Please enter your contact information.

Surname, Name

Clinical Center

Your role in the department

Address

City

Country

Email Address

Phone Number

2. Do you have access to a research office including support by a study nurse?

- No
- Yes, please enter the study nurse personal contact information below

3. Do you use hyperthermia in combination with radiotherapy or radiochemotherapy for cancer treatment in your clinical center?

- Yes
- No

4. Do you use hyperthermia in combination with chemotherapy for cancer treatment in your clinical center?

- Yes
- No

5. Which cancer sites do you treat mostly with hyperthermia combined with radiotherapy or radiochemotherapy and how many patients (N) are treated approximately in your institution per year with the respective indication?

Recurrent breast cancer, N = ______

Sarcoma, N = ______

Cervical cancer, N = ______

Rectal cancer, N = ______

Anal cancer, N= ______

Pancreatic Cancer, N= ______

Bladder Cancer, N= ______

Head and Neck, N= ______

Pediatric patients, N= ______

Others, N= ______

6. Which cancer sites do you treat mostly with hyperthermia combined with chemotherapy and how many patients are treated approximately in your institution per year with the respective indication?

Recurrent breast cancer, N = ______

Sarcoma, N = ______

Cervical cancer, N = ______

Rectal cancer, N = ______

Anal cancer, N = ______

Pancreatic Cancer, N= ______

Bladder Cancer, N= ______

Head and Neck, N= ______

Pediatric patients, N= ______

Others, N= ______

7. How many patients do you treat approximately per year using hyperthermia in combination with radiotherapy or radiochemotherapy?

______

8. How many patients do you treat approximately per year using hyperthermia in combination with chemotherapy?

9. What percentage of these patients are treated in clinical studies approximately?

10. Which hyperthermia technique are you using in your center?

- Superficial radiative
- Superficial capacitive
- Superficial infrared
- Deep Radiative
- Other (please specify)
- Capacitive
- Moderate Whole Body Hyperthermia
- Extreme Whole Body Hyperthermia

11. What is the treatment sequence applied in your clinic when patients are treated with hyperthermia in combination with radiotherapy?

- Hyperthermia followed by radiotherapy
- Radiotherapy followed by hyperthermia

12. What is the treatment sequence applied in your clinic when patients are treated with hyperthermia in combination with chemotherapy?

- Hyperthermia followed by chemotherapy
- Chemotherapy followed by hyperthermia
- Chemotherapy during hyperthermia

13. Do you measure the temperature of normal tissues and tumor tissues (when possible) during hyperthermia treatment?

- Yes
- No

14. Do you keep a record of temperature parameters (e.g. Tmax, T90, T50, T10) after treating patient with hyperthermia?

- ◯ Yes
- ◯ No

15. Do you record the time period between hyperthermia and radiotherapy/chemotherapy treatments?

- ◯ Yes
- ◯ No

16. Do you evaluate values of CEM43 from temperature patient data for each hyperthermia treatment fraction?

- ◯ Yes
- ◯ No

17. Do you extract TRISE values from temperature patient data for each hyperthermia treatment fraction?

- ◯ Yes
- ◯ No

18. What type of patient treatment plans do you create for hyperthermia in your clinical center?

- ◯ Geometry based
- ◯ Simulation based
- ◯ Both

19. What type of clinical database do you use for saving and storing patient treatment data in your clinical center? (Please write in free text)

20. Do you perform clinical follow-ups for patients treated with hyperthermia in combination with radiotherapy regularly?

- ◯ Yes
- ◯ No

21. Do you perform clinical follow-ups for patients treated with hyperthermia in combination with chemotherapy regularly?

- ◯ Yes
- ◯ No

22. If you are interested to participate in performing pooled retrospective data analysis within the HYPERBOOST project, which patient data cohorts are you willing to share in this project?

- ◯ Not interested
- ◯ All (data entered in Q5 and Q6)
- ◯ Only the following patient cohorts:
